# Brain region-specific alterations of RNA editing in PDE8A mRNA in suicide decedents

**DOI:** 10.1038/s41398-018-0331-3

**Published:** 2019-02-15

**Authors:** Fabrice Chimienti, Laurent Cavarec, Laurent Vincent, Nicolas Salvetat, Victoria Arango, Mark D. Underwood, J. John Mann, Jean-François Pujol, Dinah Weissmann

**Affiliations:** 1 ALCEDIAG/ Sys2Diag, CNRS UMR 9005, Parc Euromédecine, Montpellier, France; 2 Genomic Vision, Green Square, 80-84 rue des Meuniers, 92220 Bagneux, France; 3 Commissariat à l’Energie Atomique, Fontenay aux Roses, France; 4 Division of Molecular Imaging and Neuropathology, New York State Psychiatric Institute, New York, NY USA; 5 Department of Psychiatry, Columbia University College of Physicians and Surgeons, New York, NY USA

## Abstract

Phosphodiesterases (PDE) are key modulators of signal transduction and are involved in inflammatory cell activation, memory and cognition. There is a two-fold decrease in the expression of phosphodiesterase 8A (PDE8A) in the temporal cortex of major depressive disorder (MDD) patients. Here, we studied PDE8A mRNA-editing profile in two architectonically distinct neocortical regions in a clinically well-characterized cohort of age- and sex-matched non-psychiatric drug-free controls and depressed suicide decedents. By using capillary electrophoresis single-stranded conformational polymorphism (CE-SSCP), a previously validated technique to identify A-to-I RNA modifications, we report the full editing profile of PDE8A in the brain, including identification of two novel editing sites. Editing of PDE8A mRNA displayed clear regional difference when comparing dorsolateral prefrontal cortex (BA9) and anterior cingulate cortex (BA24). Furthermore, we report significant intra-regional differences between non-psychiatric control individuals and depressed suicide decedents, which could discriminate the two populations. Taken together, our results (i) highlight the importance of immune/inflammatory markers in major depressive disorder and suicide and (ii) establish a direct relationship between A-to-I RNA modifications of peripheral markers and A-to-I RNA editing-related modifications in brain. This work provides the first immune response-related brain marker for suicide and could pave the way for the identification of a blood-based biomarker that predicts suicidal behavior.

## Introduction

Suicide and suicidal behavior are a major public health concern. Suicide ranks among the three leading causes of death worldwide with an estimated 1 million deaths every year^1^. Suicide is a complex, multifactorial outcome whose biological basis remain poorly understood. In recent years, genome-wide association studies have contributed to a better understanding of the genetic basis of suicidal behavior. However, no single gene/SNP has been identified with genome-wide significance, though there is much evidence for an association between lower serotonergic function and suicidal behavior^2^. Rather, recent studies have shown an association between gene alterations by epigenetic mechanisms and suicidal behavior^3^. Epigenetics studies indicate a broad reprogramming of promoter DNA methylation patterns in the hippocampus of suicide decedents, including genes involved in cognitive processes^4^.

Significant differences in RNA editing of the human serotonin 2C receptor (5-HT_2C_R) in the prefrontal cortex (PFC) are reported in suicide decedents^5^. RNA editing alters the 5-HT_2C_R amino acid sequence, thereby decreasing receptor function, which may contribute to or complicate treatment in psychiatric disorders. We have recently shown that different alterations of A-to-I RNA editing of 5-HT_2c_R occur in different specific areas of the PFC^6^. Another recent study identified two polymorphisms, ADAR2 rs9983925 and 5-HT_2C_R rs6318 (Cys23Ser), as independent risk factors for suicide attempts^7^, linking genetic and epigenetic factors to elevated suicide risk.

Adenosine deaminase acting on RNA (ADAR) are enzymes that bind to double stranded RNA (dsRNA) stem loop in pre-mRNAs and convert adenosine (A) at specific positions to inosine (I) by deamination, a process termed A-to-I editing (for review see^8^). Inosine is interpreted as guanosine by the cellular machinery. RNA editing can thus induce amino acid change in coding regions or affect RNA splicing and/or stability in non-coding regions^9^. In human, consistent with mouse studies^10^, ADAR1, 2 and 3 expression have been confirmed in the brain, including prefrontal cortex and hypothalamus, with ADAR1 being by far the most expressed^11^. Deregulated expression of ADARs and abnormal editing has been observed in schizophrenia^12^ and in suicide decedents^13^. In psychiatric disorders, editing of 5-HT_2C_R has been extensively studied (for review see^14^).

In the serotonin-signaling pathway, serotonin receptors are G-protein-coupled receptors (GPCRs) involved in a variety of psychiatric disorders. The 5HT_2c_R subfamily couples with Gq/11 proteins and stimulate the activity of phospholipase C (PLC), which eventually increases intracellular Ca^2+^ levels^15^. However, 5-HT2c receptors can also couple with Gαq proteins, which in turn can also indirectly alter cyclic AMP (cAMP) levels, by either decreasing Gαs protein abundance or activating adenylate cyclase 8 (ADCY8) by the PLC/Ca2+/calmodulin pathway^16^. Additionally, 5-HT_2C_R activation can also trigger the formation of cyclic GMP (cGMP)^17^. Therefore, spatial and temporal regulation of second messenger concentration is crucial to serotonin signaling. Consequently, we focused our attention on cyclic nucleotide phosphodiesterases (PDEs). This group of enzymes, composed of 11 different families, selectively hydrolyzes cAMP, cGMP or both^18^. As such, they are key modulators of signal transduction and are involved in many normal and abnormal functions such as inflammatory cell activation, behavior, memory and cognition^19^.

In the current study, we have used a capillary electrophoresis single strand conformation polymorphism (CE-SSCP) technique, initially developed for the quantification of RNA editing of the 5HT_2C_R, to examine the complete RNA editing profile of the PDE8A mRNA in two cytoarchitectonically distinct neocortical regions in depressed suicides decedents and non-psychiatric, age- and sex-matched controls. We compared PDE8A mRNA editing in dorsolateral prefrontal cortex (DLPFC, Brodmann Area 9, BA9) and anterior cingulate cortex (ACC, Broadmann Area 24, BA24), both known to be critically involved in mood regulation and cognitive control processes^20^. We report region-specific alterations of RNA editing of PDE8A in the cortex of suicides with major depression. Analysis of different combinations of edited isoforms allowed us to discriminate between suicide and control groups, providing an immune response-related brain marker for suicide.

## Materials and methods

### Subjects

Study procedures were approved by the applicable institutional review boards. Informed consent was given by next-of-kin for tissue collection, review of relevant records and interviews for a psychological autopsy. Tissue was provided by the Human Postmortem Brain Collection at the New York State Psychiatric Institute. All cases died suddenly (see Table 1 for demographic and clinical details). Brains were collected at autopsy as described previously^6^ and kept at −80 °C until dissection^21,22^. Body fluids and brain tissue underwent toxicological screening. Individuals with a history of cerebral trauma, central nervous system disease, chronic alcoholism, illicit or therapeutic drug use or AIDS were excluded. Body fluids (blood, bile, aqueous humor and urine) were used for toxicological screening for cocaine, opiates, alcohol, antidepressants and other acidic and basic drugs. We measured pH in the cerebellum to assess the integrity of the tissue RNA as described previously^23^. At least one informant per case agreed to an interview for the purpose of a psychological autopsy, which was performed according to our previously reported method^24^; based on the SCID a DSM-IV consensus diagnostic determination was made in suicide decedents by an experienced psychiatrist (J.J.M.). Psychological autopsy results for Axis I and Axis II disorder diagnoses have been validated^24^. We used the Suicide History Form (Columbia University, New York, NY) to record past suicidal acts and a checklist for demographic and developmental data and other clinical details related to medical and psychiatric past treatment. A review of hospital records was done by an experienced psychiatrist (J.J.M.). Further details about the psychological autopsy procedure can be found elsewhere^25^. Control subjects (*n* = 8), who died from causes other than suicide, did not meet criteria for any Axis I diagnosis lifetime.

**Table 1:** Baseline characteristics of the study population

		Control (*n* = 8)	MDD (*n* = 8)	Control vs MDD *p*-value
**Main characteristics**				
Age (years)	Mean ± SE	37.4 ± 6.5	38.1 ± 6.5	0.916
	Min-Max	16–60	14–62	
Weigth (mg)	Mean ± SE	78.8 ± 8.6	79.4 ± 4.8	0.317
	Min-Max	64–137	68–110	
Post Mortem Interval (hours)	Mean ± SE	13.1 ± 2.3	18.2 ± 2.2	0.171
pH	Mean ± SE	6.6 ± 0.1	6.7 ± 0.1	0.295
RIN score	Mean ± SE	7.7 ± 0.2	7.7 ± 0.2	0.473
Sex (Male)	n (%)	8 (100.0)	8 (100.0)	NA
**Race**				
Ethnicity Caucasian, *n* (%)	*n*(%)	3 (37.5)	3 (37.5)	0.644
Ethnicity African American, *n* (%)	*n* (%)	3 (37.5)	1 (12.5)	
Ethnicity White, n (%)	*n* (%)	1 (12.5)	2 (25.0)	
Ethnicity Hispanic, *n* (%)	*n* (%)	1 (12.5)	2 (25.0)	
**Axis I**				
MDD	*n* (%)	0 (0.0)	8 (100.0)	NA
none	*n* (%)	8 (100.0)	0 (0.0)	
**Toxicology/treatments**				
Clear	*n* (%)	4 (50.0)	3 (37.5)	0.534
CO	*n* (%)	1 (12.5)	0 (0.0)	
Analgesics	*n* (%)	0 (0.0)	1 (12.5)	
Cannabinoid	*n* (%)	0 (0.0)	1 (12.5)	
none	*n* (%)	3 (37.5)	3 (37.5)	

Data are the mean ± SEM. *p*-values of main characteristics are displayed in the Wilcoxon rank-sum test. *p*-values of Races, Axis I and toxicology are displayed in chi-squared test

*CO* carbon monoxide, *MDD* major depressive disorder, *PMI* postmortem interval in hours, *RIN* RNA integrity number

### Brain regions

DLPFC and the ACC were selected because they have been consistently implicated and altered in depression and/or suicide^26^. Dissection and characterization of BA9 and BA24 areas has previously been described elsewhere^6^.

### Brain RNA isolation

Total RNA was extracted from brain specimens, purified (Qiagen RNeasy Kit; Qiagen), quantified by spectrophotometry, treated with 1 unit of DNase I (Invitrogen) for 15 min at room temperature in a final volume of 10 μl, then 1 μl of 25 mM EDTA was added and the mixture heated for 10 min at 65 °C. Total RNA was quantified by electrophoresis and the RNA integrity number (RIN) score was determined for each total RNA sample (Table 1).

### cDNA synthesis and PCR

Reverse transcription was performed using 15 units of ThermoScript reverse transcriptase (ThermoScript RT-PCR System, Invitrogen) in presence of Oligo(dT) primers at a final concentration of 2.5 μM. The first PCR reaction (final volume 25 µl, 495 bp product), was carried out on 1 µl of the reverse transcription products (or 1 ng of the editing standards) with 1 unit of Platinum Taq DNA polymerase (Invitrogen) and PDE8A intron 9-specific unlabeled primers (forward primer: 5′P-GCTGAAGCCTTCCTTCTAAGG-3′OH and reverse primer: 5′P-CCTGGGTCAACTCTAGGTCC-3′OH; final concentration 0.3 µM each). The PCR conditions were 3 min 95 °C, 35 cycles of 95 °C for 30 s, 50 °C for 30 s, and 72 °C for 30 s followed by a final elongation step at 72 °C for 2 min. Aliquots of this first PCR were checked on a 2% agarose analytic gel. The second round of PCR amplification (175 bp) was performed in a final volume of 25 µl with 1 µl of a 1:100 dilution of the first PCR products, fluorescent primers, and 1 unit of Platinum Pfx DNA polymerase (Invitrogen). The set of fluorescent, PDE8A intron 9-specific primers was as follows: forward 5′P-FAM-CTAGGGAACCCTGTTTAGTCC-3′OH (Eurofins MWG operons) and reverse 5′P- VIC-CAATGGGCACCAAAAAAGGG-3′OH (Applied Biosystems). Each primer was used at a final concentration of 0.3 µM. The PCR conditions were 4 min 94 °C, 35 cycles of 94 °C for 15 s, 50 °C for 30 s, and 68 °C for 30 s and a final elongation step at 68 °C for 2 min. Aliquots of the PCR products were checked on a 2% agarose analytic gel before CE-SSCP analysis.

### CE-SSCP analysis

Analysis of FAM and VIC-labeled cDNA fragments by the mean of their unique single-strand conformational polymorphism (SSCP) in non-denaturing polymer has been described previously^27^. Briefly, the fluorescent nested PCR products (1 µl of a 1:100 to 1:200 dilution) plus deionized formamide (11 µl) were added to a mixture of migration standards (0.5 µl). These standards were ROX-labeled PCR products from total Human Brain Cerebral Cortex RNA (Eurofins MWG operons) whose sizes covered the whole range of the retention times required for CE-SSCP analysis (see supplemental methods). After a 2 min denaturation step at 95 °C, samples were immediately chilled on ice. Non-denaturing capillary electrophoresis was carried out in an ABI PRISM® 3100-Avant Genetic Analyzer or ABI PRISM® 3130xl Genetic Analyzer (Applied Biosystems) through 80 cm-long capillaries filled with 7% “POP™ Conformational Analysis Polymer” (Applied Biosystems) and 1X TBE without glycerol. After a 3 min pre-run at 15 kV, samples were injected for 15 s at 2 kV, and electrophoresed for 105 min at 15 kV at a controlled temperature of 24 °C. Fluorescent samples were run in parallel with FAM and VIC-labeled editing standards for unambiguous identification of the different editing isoforms (an mRNA isoform is a unique molecule that may or may not contain multiple editing modifications on the same transcript, e. g., isoform BC contains a modification on both site B and site C within the same transcript). The GeneMapper v4.1.0 (AppliedBiosystems) software visualized for each sample the three electrophoregrams corresponding to FAM- and VIC-labeled sense and antisense strands as well as ROX-labeled migration standards. Quantification of each editing isoform present in a sample was performed through signal processing with a software package allowing deconvolution of isoforms standard and sample signals. Briefly, the main steps of this analysis were: (i) checking migrations standards and creation of a unique time basis, (ii) deconvolution and standardization of the editing standards areas, (iii) definition of editing standards retention times, (iv) detection of the different editing isoforms present in a sample profile and (v) relative quantification of editing isoforms by best fitting. The best fitting results yielded a specific editing profile for each sample. This profile was established by determining the percentage of non-edited and all edited isoforms present in the total analytical signal, which represents 100% in each sample. These values were used for further statistical analyses. A relative proportion of at least 0.5% was set as the threshold in order to be included in the analysis. All experiments were carried out under masked conditions and all samples from the two brain regions were assayed in the same batch for complementary DNA synthesis and PCR amplifications.

### Statistical analysis and design of the analysis

Statistical analyses on coded samples and figures were generated using the “R/Bioconductor” statistical open source software (version 3.4.3)^28^. Differences between groups were analyzed using the non-parametric Wilcoxon rank-sum test. *p*-values < 0.05 were considered as statistically significant. The calculation of the relative PDE8A isoform proportion takes into account all possible deviations between two sets of experimental conditions and is defined through the following formula:

$${\mathrm{\Delta Deviation}}_{{\mathrm{PDE}}8{\mathrm{A}}} = \% {\mathrm{Editing}}\,{\mathrm{value}}_{{\mathrm{condition}}2} - \% {\mathrm{Editing}}\,{\mathrm{value}}_{{\mathrm{condition}}1}$$

The following two criteria were considered for further detailed analysis: variations of the median value of relative proportion of RNA editing above 20% and *p*-values < 0.05 using the one-sample Wilcoxon rank-sum test, where null hypothesis H0: median variation of % editing value = 0. The sample size for this study was based on alternate editing on the 5HT_2C_R^6^.

The discriminatory power of significant PDE8A isoforms was evaluated using mROC method^29^, a dedicated program to identify the linear combination, which maximizes the AUC (Area Under the Curve) ROC. The equation for the respective combination is provided and can be used as a new virtual marker *Z*, as follows:

$${\mathrm{Z}} = {\mathrm{a}}\, \times \,{\mathrm{Isoform}}1 + {\mathrm{b}}\, \times \,{\mathrm{Isoform}}2 + {\mathrm{c}}\, \times \,{\mathrm{Isoform}}3,$$

where a, b, c are calculated coefficients and Isoform 1,2,3 are the relative proportion of individual PDE8A RNA editing level of isoform.

## Results

### Subjects

A paired case-control design was used to control demographic and assay variance. Potential confounding factors age, race of subjects, post-mortem interval, extracted tissue weight or tissue pH were not statistically different between control and MDD-suicide groups (Table 1). In order to avoid potential sex differences, only male subjects were studied. RIN values (a metric commonly used to assess RNA quality) were all >7, suggesting very limited RNA degradation, if any, during the brain collection, storage and tissue isolation procedure.

Raw data of the complete PDE8A RNA-editing profiles are shown in Supplementary Tables S1 and S2.

### Characterization of editing site combinations in intron 9 of the PDE8A pre-mRNA in human brain

**Fig. 1:**
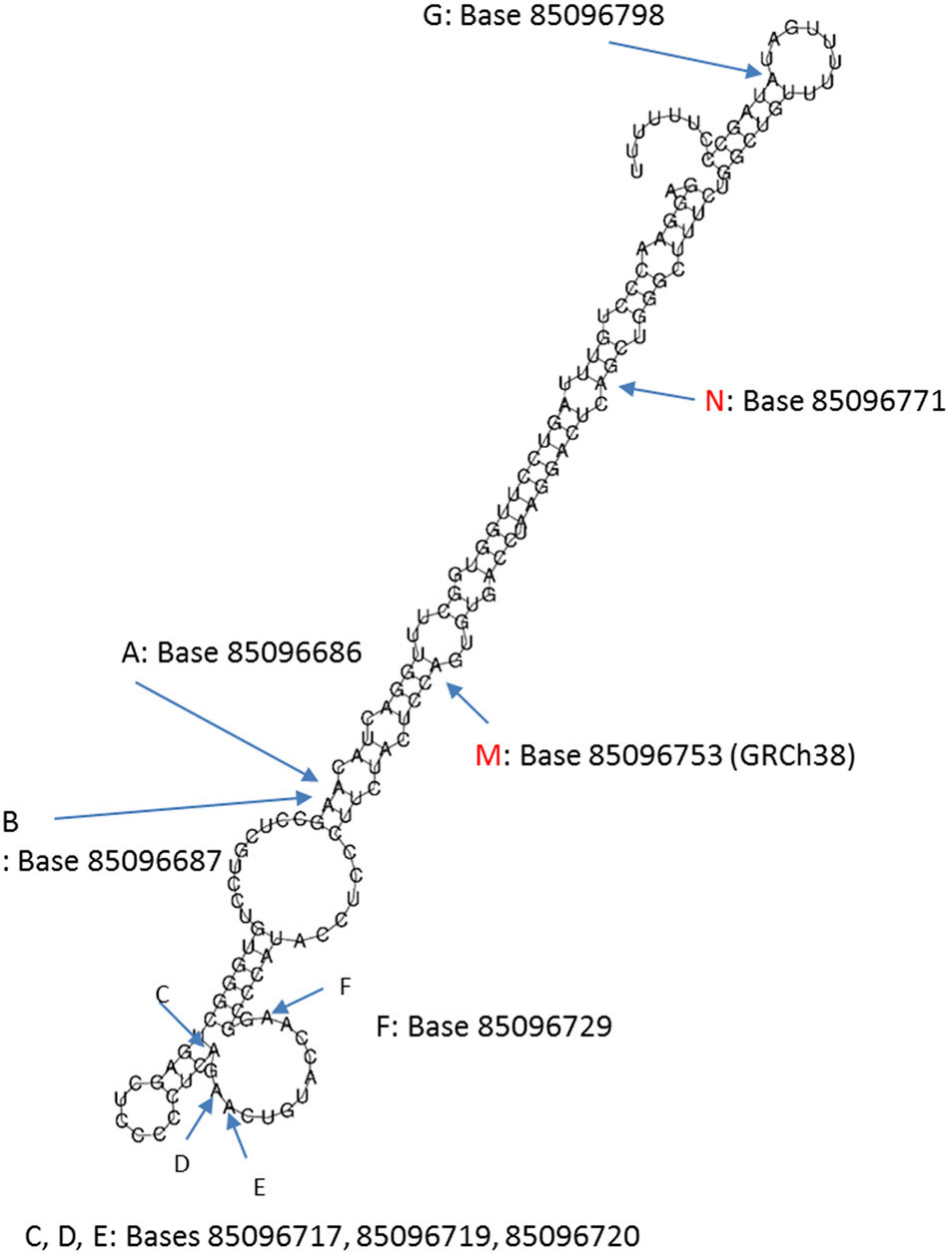
Editing sites in the intron 9 of PDE8A pre-mRNA from human brain. Putative PDE8A intron 9 mRNA secondary structure as predicted by Vienna RNA Websuite. The mRNA positions where significant editing events have been detected are highlighted with arrows. Editing Sites A to G have been previously described in T cells^32^. Newly identified sites in the brain are highlighted in red. Most of the editing sites are in close proximity to each other. All bases are annotated on chromosome 15 according to the last GRCh38 genebuild

Prediction of the secondary structure of the 175 bp sequence used in this study was performe using the Vienna RNA websuite^30^ (Fig. 1). We used the centroid structure as a representative of a set of possible structures, as the centroid of the ensemble makes 30.0% fewer prediction errors in comparison with the minimum free energy structures^31^. In the 175 bp sequence studied here in human brain, we confirmed the presence of 7 sites previously identified in T cells^32^. Moreover, we report 2 novel sites: Site M at position Chr15:85096753 (GRCh38), and site N at position Chr15:85096771 (GRCh38) (Fig. 1). The M site was edited at very low levels, e.g., <0.5%, and thus excluded from further analysis. We observed that the N site was exclusively edited in combination with A and B sites (Supplementary Tables S1 and S2), with low levels as well (<1%). For all edited sites but site D, the 5′-upstream base is either A/U or C, consistent with the preferred configuration for ADARs^33^.

### Full profile analysis of PDE8A editing in brain samples by CS-SSCP

**Fig. 2:**
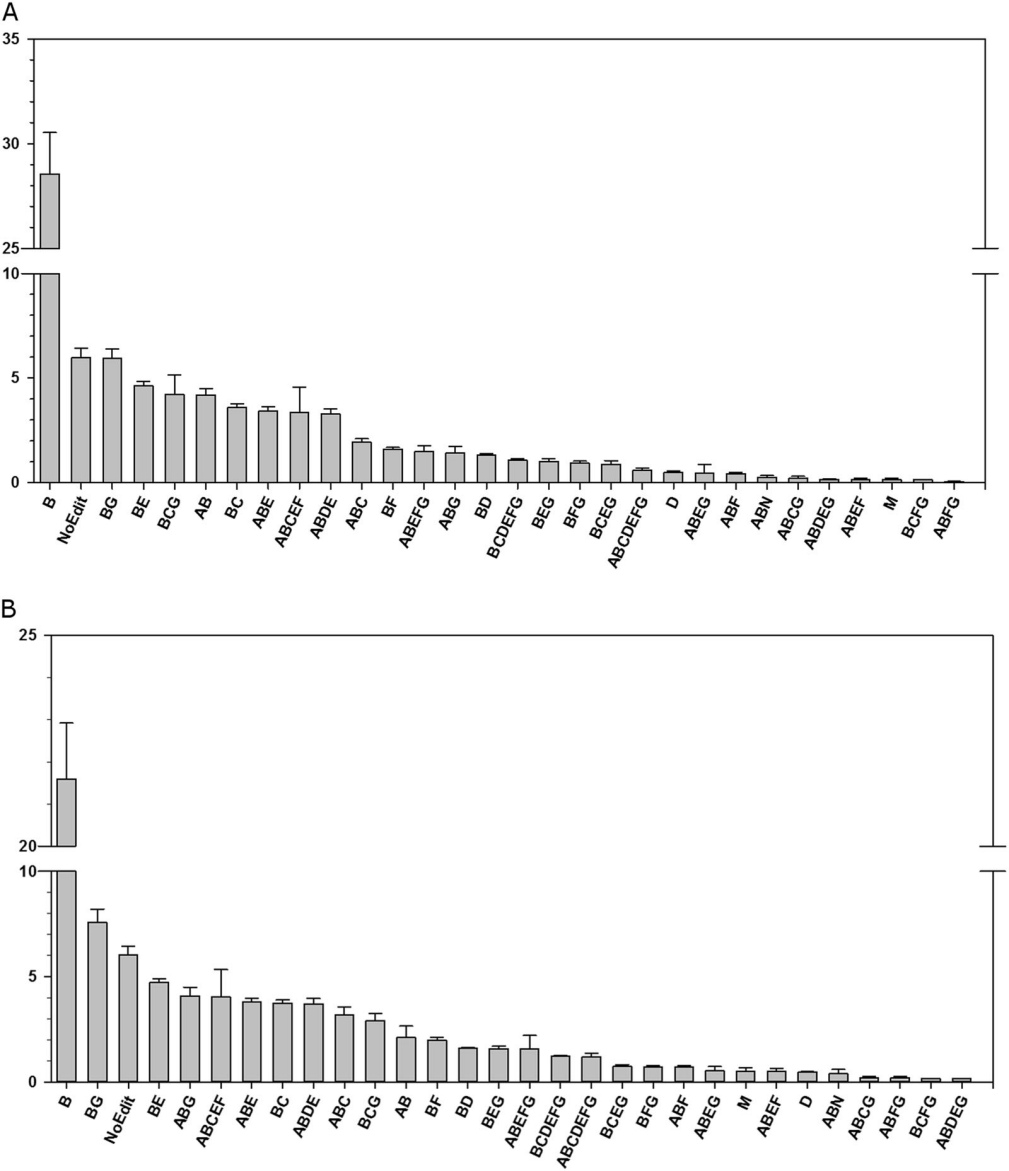
Relative isoform proportion of PDE8A mRNA in (**a**) Brodmann area 24 (BA24) and (**b**) Brodmann area 9 (BA9) measured by CE-SSCP on samples of the control group. Histograms represent relative isoform proportion (%) of the 30 detected PDE8A isoform (mean ± s.e.m.; *n* = 8). Only isoforms representing more than 0.5% of relative proportion were included in the analysis

Overall, we observed a high rate of editing in the Site 1 described in T cells^32^. However, in this site the position B (Chr15:85096687) was the most edited position, with up to 29% of isoform B in BA24 of controls (Fig. 2a) and 21.5% in BA9 (Fig. 2b). The percentage of editing on the B isoform was not affected in suicide decedents (Suppl Figure S3). In contrast, site A was only edited in combination with site B. Interestingly, the B site was edited in 27 out of the 30 isoforms identified (Figs. 2a, b). In fact, most of the isoforms identified here were in combination with the B site, suggesting that this site is preferentially targeted by ADARs. We only detected editing at the D and M sites as not occurring in combination, each with low levels of editing (Figs. 2a, b). Therefore, the B site appears to be the main site edited in all combinations isoforms identified. This is in agreement with a recent study which suggests that adjacent genomic element helps to increase the A-To-I editing efficiency^34^. In both groups, the mean cumulative relative proportion of isoforms representing less than 0.5% of the PDE8A mRNA did not exceed 2% and was found to be similar in BA9 compared with BA24 (Supplementary Fig. S3). In both BA9 and BA24, the relative proportion of the non-edited (NE) isoform was surprisingly similar at 6.0% (Fig. 2), as 94% of the isoforms were identified as being edited at at least one site. The fully edited isoform ABCDEFG was only present at low levels, e.g., 0.6% in BA24 (Fig. 2a) and 1.2% in BA9 (Fig. 2b). Editing levels of this fully edited isoforms was not changed in suicide decedents (Fig. 2 and Supplementary Fig. S3). Taken together, the current RNA-editing profile of PDE8A mRNA in brain corroborates previous data in T cells^32^ and is indicative of a high RNA-editing activity mediated by ADAR enzymes in both DLPFC and the ACC.

### Brain regional differences in PDE8A mRNA-isoform proportion

**Fig. 3:**
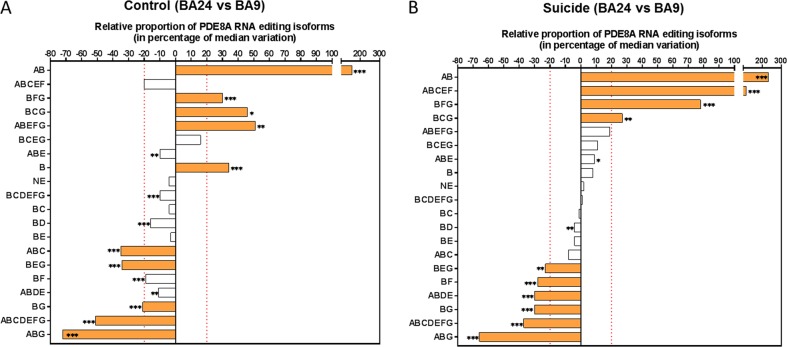
Relative proportion of PDE8A mRNA isoforms in two discrete brain regions of control (A) and suicide (B) subjects. **a** Comparison of the relative isoform proportion of PDE8A in BA24 and BA9 within the control group. The PDE8A isoforms are depicted in order of significance and abundance in BA24. **b** Comparison of the relative isoform proportion of PDE8Ain BA24 and BA9 in the suicide group. In this representation, a negative percentage from the median indicates that the relative proportion of the isoform is higher in BA9 compared with BA24. Filled bars in (**a**) and (**b**) indicate the most significant differences of the isoform proportion between the two brain structures in both control and suicide groups. Criteria for the selection are (1) a *p*-value (FDR) ≤ 0.05 using the one-sample Wilcoxon signed rank test (where null hypothesis H0: median variation = 0) and (2) a median variation ≥ ± 20%. The symbol * indicates a *p*-value (FDR) ≤ 0.05, ** indicate a *p*-value (FDR) ≤ 0.01 and *** indicate a *p*-value (FDR) ≤ 0.001

We then compared the relative proportion of PDE8A mRNA isoforms in BA24 to BA9 in non-psychiatric control subjects and suicide patients (Figs. 3a, b). Out of the 20 isoforms included in the analysis, 12 displayed a difference greater than 20% from the median between the two cortical regions in at least one group. In non-psychiatric controls subjects, the relative proportion of the AB isoform was higher in BA24 than in BA9 (156% in control group (*p* < 10^−6^), 234% in suicide group (*p* < 10^–6^), while on the opposite the isoform ABG was 72 and 66% lower (*p* < 0.001) in BA24 than in BA9 in control and suicide groups, respectively (Figs. 3a, b). Most isoforms varied in the same direction and to the same extent between non-psychiatric control subjects and suicide decedents. However, clear differences could be observed in the relative proportion of some isoforms. Notably, the most edited isoform B was significantly higher (34%, *p* < 0.001) in BA24 of controls but was not significantly different between BA24 and BA9 in suicide decedents (Figs. 3a, b). Likewise, isoform ABEFG was 51% higher in BA24 than in BA9 of controls (*p* < 0.005) but was not significantly different in suicide decedents between the two cortical regions. Conversely, isoform ABDE was 11% higher in BA24 of controls (*p* < 0.01), and 30% less (*p* < 0.001) in BA9 of suicide decedents. The distribution of isoform ABCEF was comparable in BA24 *vs*. BA9 of controls, but was significantly higher (115%, *p* < 0.0001) in BA24 compared with BA9 in suicide decedents.

### Suicide-induced specific changes in PDE8A mRNA editing

**Fig. 4:**
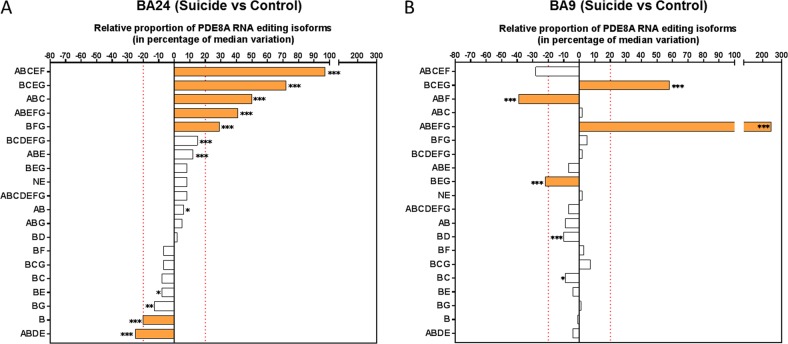
Brain regional specificity of PDE8A mRNA editing changes in suicide with major depression. **a** Comparison of the relative isoform proportion of PDE8A in control and suicide group within BA24 area. The PDE8A isoforms are depicted in order of significance and abundancy in suicide group. A negative value indicates relative decrease of the isoform whereas an increase in the relative proportion is indicated by a positive value. **b** Comparison of the relative isoform proportion of PDE8A in control and suicides within the BA9 area. Filled colored bars in (**a**) and (**b**) indicate most significant differences between the control and suicide groups. Criteria for the selection are (1) a *p*-value (FDR) ≤ 0,05 using the one-sample Wilcoxon signed rank test (where null hypothesis H0: median variation = 0) and (2) a median variation ≥ ± 20%. The symbol * indicate a *p*-value (FDR) ≤ 0.05, ** indicate a *p*-value (FDR) ≤ 0,01 and *** indicates a *p*-value (FDR) ≤ 0.001

We next analyzed the relative mRNA-editing profile of PDE8A in both non-psychiatric controls and depressed suicide decedents by measuring PDE8A isoforms specifically in each cortical area (Fig. 4). In BA24, we identified five isoforms (ABCEF, BCEG, ABC, ABEFG and BFG) significantly upregulated (*p*-value(FDR) < 0.001) in depressed suicide decedents, with a difference greater than 20% from the median between the two groups (Fig. 4a). Of these 5 isoforms, isoform ABCEF displayed the greatest difference, with editing values in suicide decedents almost double those of controls (Fig. 4a). Isoforms BCDEFG and ABE were also (*p*-value (FDR) < 0.001) upregulated in suicide decedents in BA24 though they did not reach the 20% increase threshold. Isoforms B and ABDE medians were downregulated more than 20% in suicides compared to controls in BA24 (*p*-value (FDR) < 0.001) but not in BA9 (Fig. 4b). Overall, we identified 12 isoforms in BA24 with differential regulation (*p*-value (FDR) ≤ 0.01) between MDD-suicide and control groups (Fig. 4a). On contast, the same analysis in BA9 identified only 6 isoforms with differential regulation (*p*-value (FDR) ≤ 0.01) between the two groups (Fig. 4b). Isoform ABEFG was the isoform with the greatest upregulation in MDD-suicide in BA9 (>200% increase, p(FDR) < 0,001). Notably, this isoform was also increased, to a lesser extent, in the BA24 of suicides, suggesting that RNA editing affects this isoform in different cortical areas. Interestingly, isoform ABN, which includes a novel site described in this work, was higher in suicide in both BA9 and BA24 (Supplementary Tables S1 and S2). However, we didn′t take this isoform into consideration in further analyses due to low levels of editing (<0.5%) in controls. Of note, isoform B expression, which was downregulated in BA9 *vs*. BA24 in non-psychiatric controls (Fig. 3a) and downregulated in BA24 in the MDD-suicide group (Fig. 4a), was not different between the groups in BA9 (Fig. 4b). Conversely, isoform BEG was significantly downregulated in MDD-suicide in BA9 only.

**Fig. 5:**
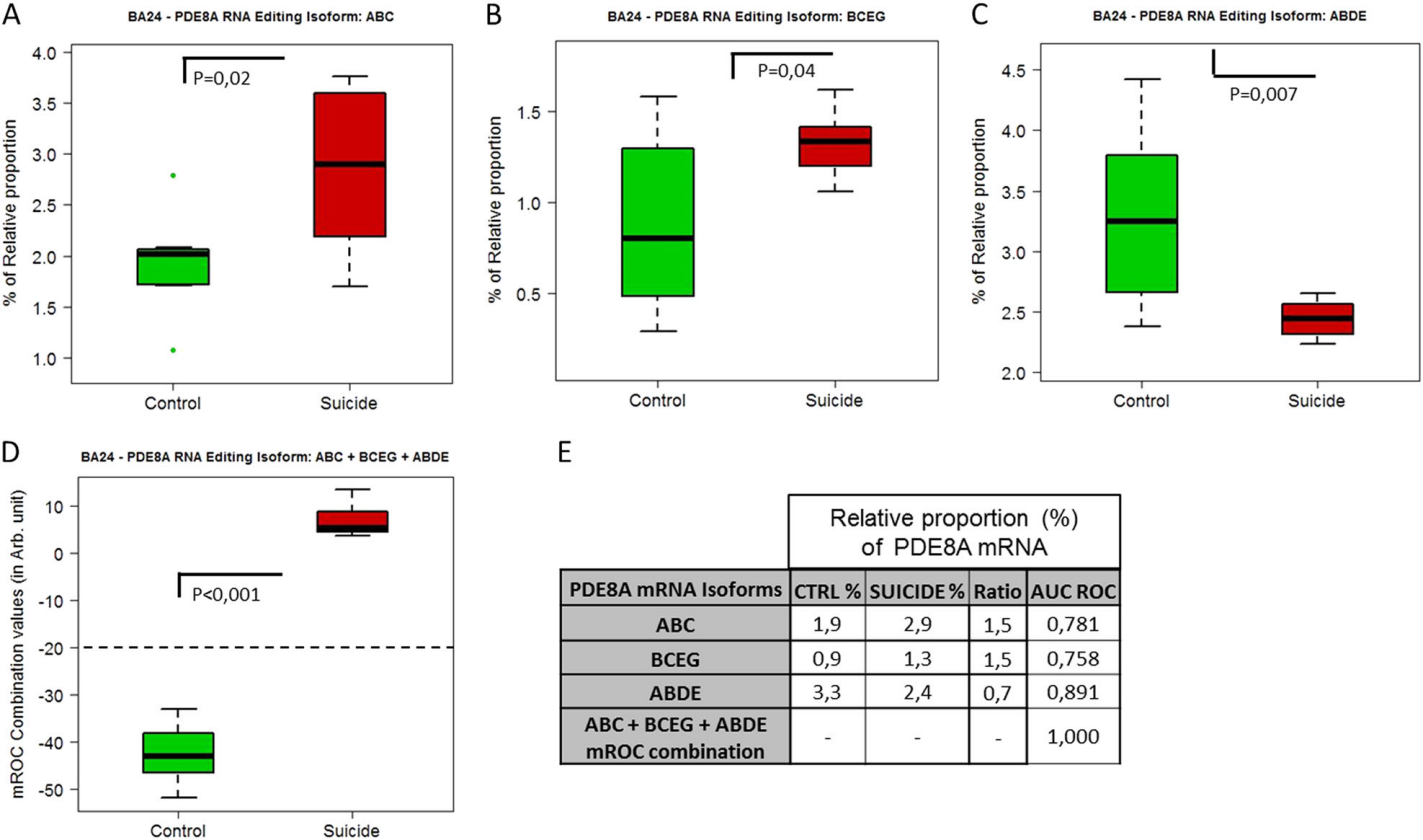
Suicide-induced alterations of the relative proportion of PDE8A isoforms in BA24. **a**–**c** Comparison of the relative proportion of ABC, BCEG and ABDE isoforms in control (*n* = 8) and suicide groups (*n* = 8). Boxplot represents the distribution of the relative proportion of PDE8A mRNA in BA24 of both groups. **d** Boxplot representation of mROC combination of relative proportions (%) of ABC + BCEG + ABDE PDE8A isoforms in BA24 of both groups. **e** Table showing the most significant changes of the relative proportion of PDE8A mRNA isoforms in BA24 of both groups. The symbol * indicates, for Wilcoxon rank-sum test, a *p*-value ≤ 0.05, ** indicates a *p*-value ≤ 0.01 and *** indicate a *p*-value ≤ 0.001

**Fig. 6:**
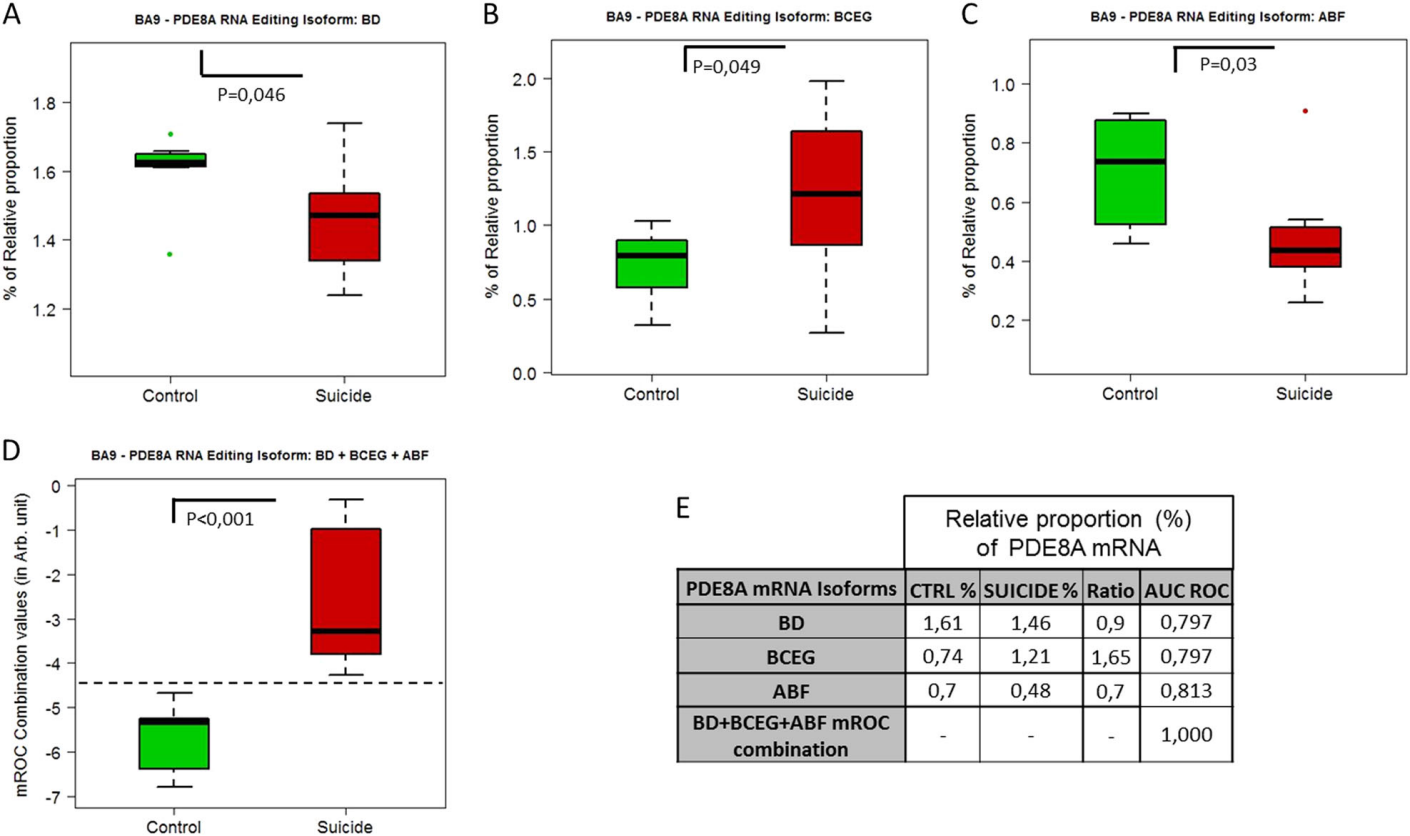
Suicide-induced alterations of the relative proportion of PDE8A isoforms in BA9. **a**–**c** Comparison of the relative proportion of BD, BCEG and ABF isoforms in control (*n* = 8) and suicide groups (*n* = 8). Boxplot represents the distribution of the relative proportion of PDE8A mRNA in BA9 of both groups. **d** Boxplot representation of mROC combination of relative proportions (%) of BD + BCEG + ABF PDE8A isoforms in BA9 of both groups. **e** Table showing the most significant changes of the relative proportion of PDE8A mRNA isoforms in BA9 of both groups. The symbol * indicates, for Wilcoxon rank-sum test, a *p*-value ≤ 0.05, ** indicate a *p*-value ≤ 0.01 and *** indicate a *p*-value ≤ 0.001

Next, we studied the relative proportion of specific isoforms between suicide and controls, alone or in combination, in each cortical area, and tested both specificity and sensibility by the Receiver Operating Characteristic (ROC) curve to assess their potential as diagnostic tests. In BA24, isoforms ABC, BCEG and ABDE were found to be altered in suicide (Figs. 5a–c) and discriminated between controls and suicides, each displaying an AUC ROC > 0.75 (Fig. 5e). Furthermore, when used in combination, these isoforms were found to be highly discriminative between the control and MDD-suicide populations, with an AUC ROC = 1 (Figs. 5d, e). In BA9, the same analysis with isoforms BD, BCEG and ABF yielded the same performance, each displaying and AUC ROC > 0.79 (Figs. 6a–c, e). When combined, these isoforms were highly discriminative and reached an AUC ROC = 1 (Figs. 6d, e).

## Discussion

In this study, we used CE-SSCP to study the mRNA editing profile of cyclic nucleotide phosphodiesterase 8A in two brain regions from suicide decedents and normal controls. We performed full editing profile analysis of PDE8A editing and found two novel sites in intron 9 editing island. PDE8A mRNA editing profiles in dorsolateral prefrontal cortex (BA9) and anterior cingulate cortex (BA24) differed and there were region-specific alterations of RNA editing of PDE8A in suicide decedents with major depression. An altered pattern of isoforms of edited PDE8A are an immune response-related brain marker for suicide, as such combinations clearly separated the controls and MDD suicide groups.

We previously used the same CE-SSCP methodological approach to analyze the changes induced in 5-HT_2C_R mRNA-editing levels (i) by interferon treatment in SH-SY5Y human neuroblastoma cell line^27,35^ and (ii) in the cortex of suicide decedents with major depression^6^. Using this technique, we confirmed editing sites previously described in white blood cells^32^. Orlowski and colleagues used traditional cloning-sequencing approach to detect RNA editing in PDE8A transcripts in T cells of control and systemic lupus erythematosus patients^32^. A subsequent study used direct sequencing of RT-PCR products from U-397 and THP-1 cell lines products to investigate RNA editing signature during myeloid leukemia cell differentiation^36^. With CE-SSCP technology, integration of the individual electrophoresis signals and the small variation over its 11,000 points, which characterized the analytical CE signal for each group of samples and from each cortical region provides highly accurate signals. Moreover, when combined with the best-fitting process of each individual signal, this technique accurately measures the relative proportion of all identified PDE8A isoforms within each sample, thereby allowing direct comparison of the editing level between samples. Furthermore, studying conformational isoforms of the RNA can indicate the presence of editing inducer elements, which might also be edited at different sites and contribute to the efficient editing at distant specific sites. Assaying isoforms provides a more comprehensive picture than separate assays of editing levels at different sites, as editing in one region can significantly influence editing in proximal sites^34^. Moreover, the sensitivity of CE-SSCP has enabled us to identify two novel editing sites at PDE8A intron 9 in the brain, suggesting the presence of an editing island, i.e., cluster of editing sites in close proximity to each other, in intron 9 of PDE8A. Remarkably, while this non-coding region of intron 9 is highly edited at multiple specific sites, the level of non-edited (NE) isoform was relatively stable in both brain areas and conditions, thereby suggesting that the editing events observed in all other isoforms are the result of specific enzymatic activity of ADARs.

We found significant regional differences in PDE8A mRNA editing between BA9 and BA24. It is established that RNA transcript editing can differ across regions of the brain^37^. A-to-I RNA editing is crucial for normal brain function and regulates key neurotransmitter receptors in the brain, including glutamate and serotonin receptors^38^. RNA editing signatures from single-cell transcriptomics revealed that only a few sites located in genes for glutamate receptors, were edited in most neurons^39^. RNA editing levels of most ionotropic glutamate receptors appear to be finely modulated in different brain areas^40^. A whole transcriptome RNA-Seq study in human brain identified differential RNA editing in 8 genes, including GRIK2 and GRIA2, across 9 brain regions^41^. Another example where RNA editing mediates fine-regulation of neurotransmission is a specific isoform of tryptophan hydroxylase, namely TPH2^42^. While TPH1 is mostly a non-neuronal enzyme, TPH2 encodes a brain-enriched isoform of tryptophan hydroxylase, which is the rate-limiting enzyme in the biosynthesis of serotonin (5-HT). Two TPH2 splice variants are dynamically RNA-edited in a mutually exclusive manner, and RNA editing of both TPH2 isoforms leads to protein variants with distinct catalytic properties^42^. Variants of both TPH2 and ADAR2 (also known as ADARB1) have been linked to increased predisposition to suicide attempt in psychiatric patients exposed to an adverse childhood environment^43^. Based on the above, it is reasonable to assume that regionally differential PDE8A mRNA editing might be present across brain regions.

We identified region-specific alterations of RNA editing of an immune response marker, PDE8A, in the cortex of suicide decedents with major depression. An association between inflammation and depression has been proposed following examination of both blood and brain^44^. Gene expression profiling in postmortem prefrontal cortex of MDD patients showed altered expression of CNR2, a cannabinoid receptor that has been implicated in immune and inflammation responses^45^. A subset of gene expression biomarkers previously identified in suicidality have biological roles in immune and inflammatory response^46^. Subsets of patients who suffer from depression have elevations in circulating pro-inflammatory cytokines TNF-α, IL-1β, and IL-6^47^. Moreover, postmortem immunohistochemical analysis of brain tissue from suicide decedents, including dorsolateral prefrontal cortex and anterior cingulate cortex, showed increased activation of microglia, which secrete neuroendocrine factors and cytokines^48^. A recent meta-analysis suggests that common genetic variants and gene-expression pathways are involved in both immune activation and depression^49^. In fact, there is an increasing body of evidence in support of an activation of the inflammatory system in the pathophysiology of MDD; gene expression studies have shown an increased expression in genes related to immune and inflammatory response in the brain of MDD and suicide decedents^44,46,50–52^. However, most studies focus on gene expression and do not take RNA editing into account. We previously identified region-specific alterations in editing of 5HT_2C_R, whose loss of function is likely to contribute to the physiopathology of major depressive disorder^6^. Interestingly, the reported alterations of PDE8A RNA editing in the cortex of suicide decedents with major depression were observed in a genomic position, e.g., 15q25.3, which was reported to contain genes that contribute to susceptibility to major depression. Indeed, genome-wide suggestive evidence for non sex-specific linkage to MDD was observed on chromosome 15q25-q26^53^. Subsequent fine mapping of the 15q region demonstrated further evidence of linkage^54^.

Editing of PDE8A was initially identified in T-cells in systemic lupus erythematosus, a chronic autoimmune disorder^32^. Recent data suggest that the brain is directly influenced by peripherally derived chemokines and cytokines and that some immune cells can actually access the brain and thereby influence neuronal networks, which malfunction in depression^55^. Indeed, it appears that chronic low grade inflammation can damage neuronal networks, at least in part through activation of the hypothalamic pituitary axis, activation of both peripheral macrophages and the tryptophan-kynurenine pathway (for review see^56^). Many different phosphodiesterase isoforms are expressed in neurons and glia; mutations in PDEs are likely to be associated with several diseases of the CNS^18^. More specifically, in the same neuron, cAMP compartmentalization and control of cyclic nucleotide levels within narrow ranges of concentrations are ensured by diverse PDE subtypes^57^. Hence, cyclic nucleotides play a key role in inter/intracellular communication in neurons and affect neurotransmission in psychiatric disorders^58^.

Among the PDEs genes, all of which are expressed at different levels within discrete sub-regions of the human brain, it has been shown that PDE8A mRNAs were twofold less expressed in the temporal cortex of MDD patients when compared to controls^59^. The PDE8A pre-mRNA is the post transcriptional substrate of RNA editing by the ADAR enzymes^60^. The exact role and function of RNA editing in intron 9 of PDE8A deserves to be further elucidated. Measurement of expression levels of ADARs in the brain of major depressive disorder/suicide decedents and examination of both expression and editing in PDE8A would be interesting. Greater ADAR1 expression is reported in the dorsolateral prefrontal cortex of major depressive suicide decedents^13^, but the authors did not study phosphodiesterases. ADAR1 is upregulated by Type I interferon^27^, which is known to regulate the activity of the human immune system. Treatment of normal T cells with IFN-α significantly modified RNA editing in PDE8A intron 9^32^. Interferon treatment is known to induce depressive symptoms and major depression in a considerable number of subjects^61^. Patients with chronic hepatitis C virus infection who developed psychiatric symptoms under antiviral combination therapy with IFN-α and ribavirin have altered PDE8A editing (Weissmann et al, manuscript in preparation). A central role of RNA editing and non-coding RNAs in brain homeostasis (for review see^62^) raises the possibility that RNA editing at PDE8A intron 9 might have regulatory functions after splicing, which are yet to be identified.

The observation of region-specific alterations of RNA editing of PDE8A in the cortex of suicide decedents with major depression raises the possibility of providing a marker for discriminating between suicides and control groups and suggests an immune response-related brain mechanism for suicide. Such data should be interpreted with caution and confirmed in a larger scale. Another limitation of the study is also that we focused on changes in RNA editing in PDE8A, though it is conceivable that modifications in RNA editing on other markers could also occur, as it has been already observed for 5HT_2C_ receptor^6^. Given the possibility that altered PDE8A mRNA editing appears to be related to suicide risk, It would also be important to validate our results in other tissues, e.g., blood samples, and in a larger cohort, possibly including nonfatal suicide attempters. As PDE8A can be detected in white blood cells, the link between PDE8A mRNA editing and suicide could pave the way for predictive RNA-editing-based blood biomarkers measuring the risk of depressive symptoms and suicide.

## Supplementary information

Figure S3

Figure S4

Table S1

Table S2

Supplemental Figures legends and methods
