# Correction: Brain region-specific alterations of RNA editing in PDE8A mRNA in suicide decedents

**DOI:** 10.1038/s41398-019-0453-2

**Published:** 2019-03-14

**Authors:** Fabrice Chimienti, Laurent Cavarec, Laurent Vincent, Nicolas Salvetat, Victoria Arango, Mark D. Underwood, J. John Mann, Jean-François Pujol, Dinah Weissmann

**Affiliations:** 1 ALCEDIAG/ Sys2Diag, CNRS UMR 9005, Parc Euromédecine, Montpellier, France; 2 Genomic Vision, Green Square, 80-84 rue des Meuniers, 92220 Bagneux, France; 3 Commissariat à l’Energie Atomique, Fontenay aux Roses, France; 4 Division of Molecular Imaging and Neuropathology, New York State Psychiatric Institute, New York, NY USA; 5 Department of Psychiatry, Columbia University College of Physicians and Surgeons, New York, NY USA

**Correction to: Translational Psychiatry**

10.1038/s41398-018-0331-3 Published online 15 Feb 2019.

Author forgot to attach a supplementary doc file which includes the supplementary methods and supplementary figure legends.

