# Supplementary figures and images for: Brain region-specific alterations of RNA editing in PDE8A mRNA in suicide decedents
Source: Transl Psychiatry. 2019 Feb 15;9:91. doi: 10.1038/s41398-018-0331-3 (PMC6377659; doi:10.1038/s41398-018-0331-3)

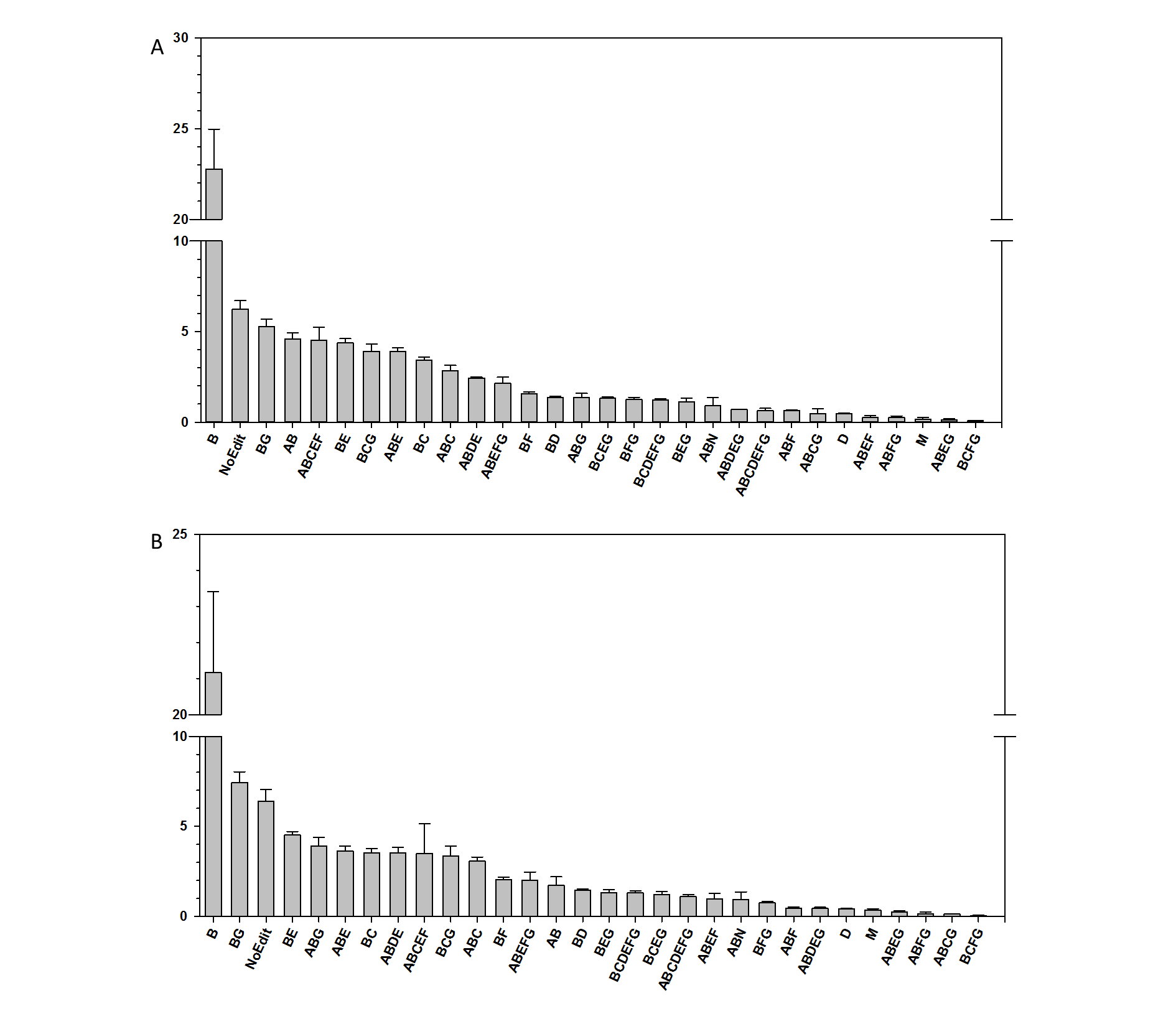

Supplement: Supplementary file 1 — Figure S3 [file 41398_2018_331_MOESM1_ESM.tif]

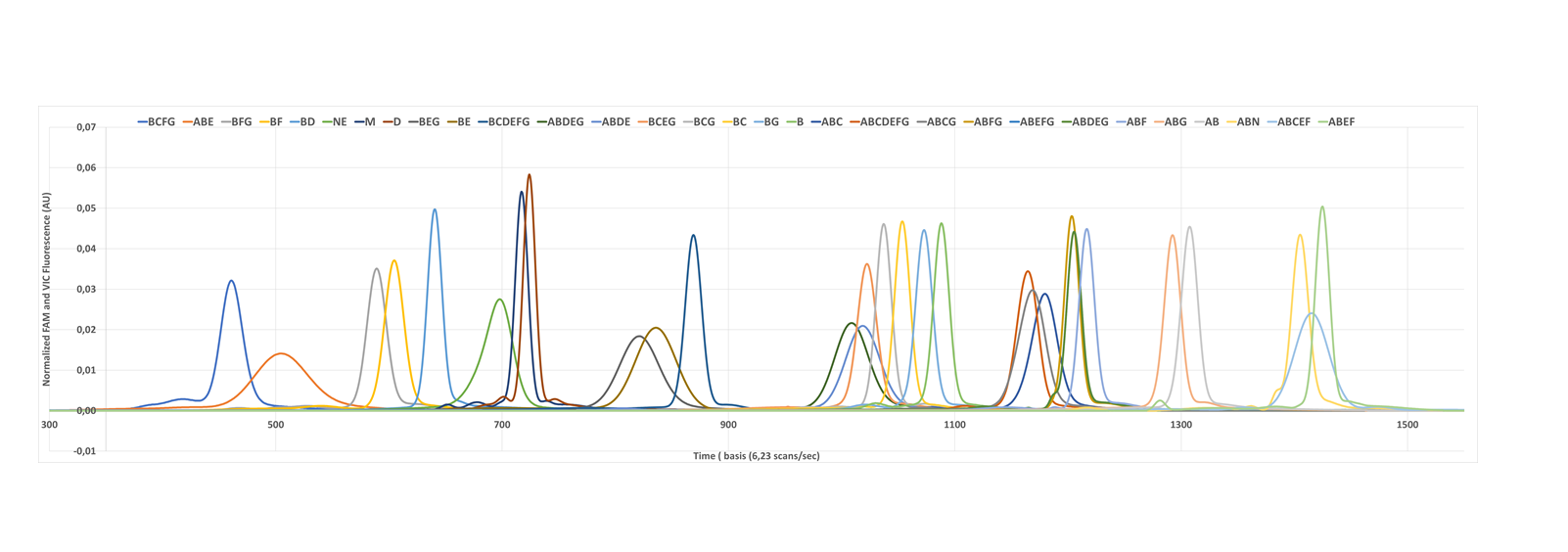

Supplement: Supplementary file 2 — Figure S4 [file 41398_2018_331_MOESM2_ESM.tif]
