# Supplementary material for: Brain region-specific alterations of RNA editing in PDE8A mRNA in suicide decedents
Source: Transl Psychiatry. 2019 Feb 15;9:91. doi: 10.1038/s41398-018-0331-3 (PMC6377659; doi:10.1038/s41398-018-0331-3)
Supplement: Supplementary file 5 — Supplemental Figures legends and methods [file 41398_2018_331_MOESM5_ESM.docx]

**Supplemental Figures legends**

**Supp Table S1.** Complete raw data set (n=8) from CE-SSCP analysis of PDE8A mRNA editing relative proportions (%) in BA24 in control (A) and Suicide (B) male subjects.

**Supp Table S2.** Complete raw data set (n=8) from CE-SSCP analysis of PDE8A mRNA editing relative proportions (%) in BA9 in control (A) and Suicide (B) male subjects.

**Supp Figure S3.** Relative isoform proportion of PDE8A mRNA in (A) Brodmann area 24 (BA24) and (B) Brodmann area 9 (BA9) CE-SSCP on samples of the suicide group. Histograms represent relative isoform proportion (%) of the 30 detected PDE8A isoform (means ± s.e.m.; n =8). Only isoforms representing more than 0.5% of relative proportion were included in the analysis.

**Supp Figure S4.**Electrophoretic profile of migration standards as separated by CE-SSCP. The normalized FAM and VIC fluorescence is plotted against the number of scans.

**Supplemental methods**

Construction of migration standard for CE-SSCP

1 µg of total RNA (Human Brain Cerebral Cortex Total RNA, Clontech, ref 636561 [pool of 10 male/female Caucasians, ages: 20-68y) was treated with 1 unit of RQ1 RNase-free, DNase (Promega, ref M610A) for 30 minutes at 37°C. The reaction was stopped by adding 1µl of Stop Solution (20mM EGTA, Promega), heated for 10 minutes at 65°C and immediately placed and kept on ice. DNase-treated RNAs were then reverse transcribed with the Thermoscript RT-PCR system Plus Taq (Invitrogen) and the gene-specific primer PDE8A-RT: 5’P-GTGGTAGGGAAAGCCAGGATG-3’OH located in intron 9 of the Human *PDE8A* gene. The PCR reaction (final volume 50µl) resulting in a 202 bp fragment, was carried out on 2µl of the reverse transcription products with 1 unit of Platinum *Pfx* DNA polymerase (Invitrogen) and intron 9-specific primers (forward primer : 5’P-CAACCCACTTATTTCTGCCTAG-3’OH and reverse primer : 5’P- TTCTGAAAACAATGGGCACC-3’OH; final concentration 0,3µM each). After a denaturing step at 95°C for 5 minutes, the PCR was brought to its final point after 35 cycles (30 seconds at 95°C; 30 seconds at 62°C with a decreasing temperature after cycle 10 by 0,5°C every 1 cycle, and 30 seconds at 68°C), and a final elongation step of 2 minutes at 68°C. Aliquots (5µl) of the amplification products were used to check the quantity and the quality of amplicons on a 2% agarose analytic gel. The remaining 45µl of each PCR reaction were run on a preparative 2% agarose gel. Under longwave UV light, agarose slices containing the PCR products were cut off and DNA was then purified with the QIAquick gel extraction kit (Qiagen, ref 28704). The purified PCR products were sent to GeneCust for cloning in the pUC57 vector, and sequencing. One hundred and fifty clones coming from cerebral cortex were sequenced. Sequence analysis was performed and the occurrence of each editing isoform quantified. Plasmids corresponding to the different editing isoforms were then amplified and used as standards in CE-SSCP experiments.
